# Comparison of *in vitro* fertilization outcomes in ICSI cycles after human sperm preparation by density gradient centrifugation and direct micro swim-up without centrifugation

**DOI:** 10.5935/1518-0557.20170022

**Published:** 2017

**Authors:** Simone Palini, Silvia De Stefani, Mariangela Primiterra, Serena Benedetti, Stefano Barone, Luca Carli, Enrico Vaccari, Ulug Murat, Wilfried Feichtinger

**Affiliations:** 1 IVF Unit, Cervesi Hospital Cattolica, Cattolica (RN), Italy; 2 Section of Clinical Biochemistry and Molecular Genetics, Department of Biomolecular Sciences, University of Urbino Carlo Bo, Urbino (PU), Italy; 3 IVF Unit, Versilia Hospital, Lido di Camaiore (LU), Italy; 4 Wunschbaby Zentrum Feichtinger, Wien, Austria; 5 IVF Unit, Ota Jinemed Hospital, Istanbul, Turkey

**Keywords:** Sperm treatment, centrifugation, swim-up, pregnancy rate, blastulation rate, DNA damage

## Abstract

**Objective:**

The aim of this study was to evaluate the efficacy of a non-expensive, easy and fast technique (direct micro swim-up) for sperm preparation in intracytoplasmic sperm injection (ICSI) treatments without the use of centrifuge.

**Methods:**

We carried out a multicentric study in which a total of 140 ICSI-cycles were included. Sibling oocytes were divided into two groups according to semen preparation procedures: group A, discontinuous gradients (DG) (oocytes n=668), and group B, direct micro swim-up (MSU) (oocytes n=660). We analyzed differences in some key performance indicators.

**Results:**

Fertilization rates were not statistically different between the DG and MSU groups (76.0% vs. 81.8%, respectively, *p*=0.248); while significant differences were found in blastulation rates per fertilized oocytes (41.7% vs. 58.5%, *p*=0.009), blastulation rates per D3 embryos (46.1% vs. 63.7%, *p*=0.045), and pregnancy rates (25.8% vs. 41.9%, *p*=0.045). The abortion rate was reduced in the MSU group as compared to DG, but not in a significant manner (12.9% vs. 29.4%, *p*=0.161).

**Conclusion:**

The MSU procedure has the advantage of reducing costs, time and mismatches, while ensuring comparable, and in some cases, better results than DG treatments. This technique can therefore be used as an alternative method to other conventional semen treatments.

## INTRODUCTION

Many methods have been developed for semen sample preparation for *in vitro* fertilization (IVF) treatments according to semen quality parameters (concentration, motility, morphology). The procedures include, as major techniques, simple washing, direct swim-up, and discontinuous density gradients (World of Health Organization, 2010).

To perform these procedures, a laboratory must be equipped with specific materials and media that increase the costs of the entire IVF treatment. Moreover, all the procedures require centrifugations varying in speed and time, and the movement of the samples from one tube to another thus increasing the risk of mismatches.

A wide variability in the techniques used for semen treatments leads to the need to evaluate the potential benefits and risks of each procedure, in terms of (i) sperm function, embryo development and treatment outcome; (ii) technical complexity and risk of sample mismatches; (iii) material costs and time of the procedure.

Regarding sperm function maintenance following the two most conventional methods of sperm preparation, namely swim-up (SU) and discontinuous gradients (DG), there are different opinions in the literature. Some authors reported a higher recovery rate of total motile, progressive motile and viable sperm cells after DG than after SU (^Ding *et al.*, 2000^; ^Ricci *et al.*, 2009^). Other benefits of DG are the higher percentage of morphologically normal spermatozoa (^Prakash *et al.*, 1998^; ^Hammadeh *et al.*, 2001^) and the lower deformity rate and DNA fragmentation index (^Xue *et al.*, 2014^). In contrast, other authors reported that the proportion of fast spermatozoa was enhanced in SU preparations (^Chantler *et al.*, 2004^) and that spermatozoa reported less vacuolizations (^Monqaut *et al.*, 2011^).

Another topic of debate concerns the use of the centrifuge and the formation of reactive oxygen species (ROS) during semen treatment. DG centrifugation may be detrimental to sperm DNA integrity, especially in semen samples from infertile men with impaired motility and morphology (^Zini *et al.*, 2000^). These samples generate high pathological ROS levels (because of morphologically abnormal sperm) and have a lower total antioxidant capacity (TAC) than seminal plasma from fertile men (^Benedetti *et al.*, 2012^). Consequently, serial centrifugations can result in augmented ROS generation and further loss of sperm function, probably due to the enhanced DNA denaturation during centrifugation.

To assess the best procedure for semen treatment, one must also consider the time of centrifugation. Some authors (^Shekarriz *et al.*, 1995^) reported that the time of centrifugation is more important than g-force for inducing ROS formation in semen, recommending a shorter centrifugation time in the preparation of sperm for assisted reproductive techniques.

Concerning pregnancy rates in intracytoplasmic sperm injection (ICSI) cycles, some authors reported values of 46.2% and 57.1% for SU and DG, respectively (^Borges *et al.*, 2013^), while other papers reported rates of 21.1% and 33.3% (^Van der Zwalmen *et al.*, 1991^) or 33.3% and 32.8% (^Hammadeh *et al.*, 2001^), respectively. These results do not show a conclusion regarding fertilization and pregnancy rates in SU and DG procedures.

In this study, we used a simple, fast and non-expensive technique for semen preparation in ICSI cycles, namely direct micro swim-up (MSU), as recently reported (^Palini *et al.*, 2016^). MSU is a safe treatment regarding the presence of microbiological contaminants; indeed, we demonstrated that sample fractions obtained after the MSU procedure and used for ICSI insemination were free of contaminants even in semen samples positive for sexual pathogens (^Palini *et al.*, 2016^). Despite this evidence, inclusion criteria for the present study were negative urethral and vaginal swabs, confirmed before treatment, and negative infection tests (HIV, HCV, HbsAg, HbcAb, VDRL-TPHA).

The aim of the present study was to compare MSU with DG procedures in terms of fertilization rates (FR), blastulation rates (BR), pregnancy rates (PR), and abortion rates (AR).

## MATERIALS AND METHODS

This is a multicentric study involving four Assisted Reproduction Clinics in Europe. The study was approved by an Internal Review Board (IRB) of each participating center. All the four clinics used the same media, procedures and incubators.

We retrospectively evaluated 140 ICSI cycles performed from October 2014 to June 2015 involving 140 couples (women aged 35±4 years, men aged 38±3 years) undergoing IVF treatments due to male (n=52), female (n=20), multifactorial (n=8), or idiopathic (n=60) infertility. The inclusion criteria were as follows: negative urethral and vaginal swabs confirmed before treatment, negative infection tests (HIV, HCV, HbsAg, HbcAb, VDRL-TPHA) for couples, at least 8 mature oocytes retrieved at pickup, oocyte insemination with ICSI technique, only fresh elective single embryo transfer (eSET) at blastocyst stage, and sperm concentration ≥1x10^6^/ml.

**Figure 1:**
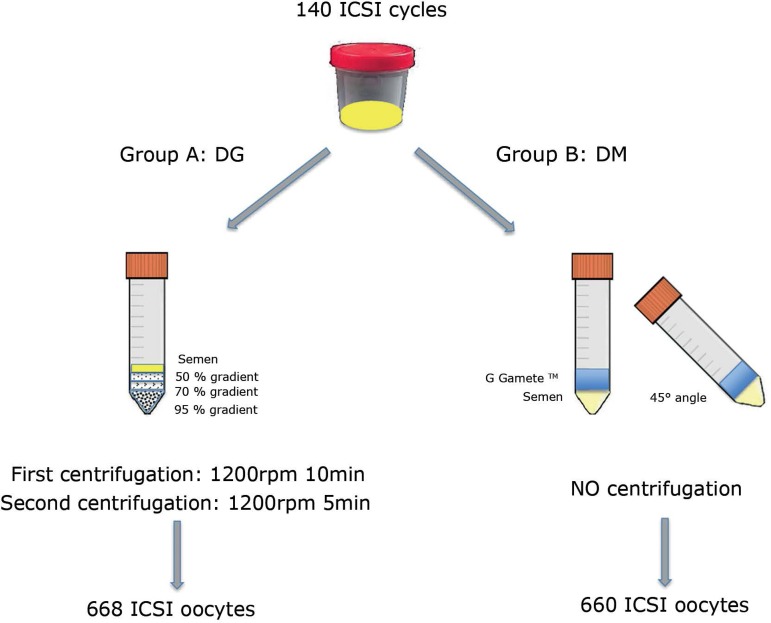
Study design.

In each cycle, we divided sibling oocyte populations into two groups according to the technique used for sperm preparation for the ICSI: group A, discontinuous gradients (DG), and group B, direct micro swim-up (MSU) (Figure 1).

The study was carried out in accordance with the Code of Ethics of the World Medical Association (Declaration of Helsinki) for experiments involving humans following the Internal Review Board's approval (granted September 2014).

### Semen sample collection

All semen samples were collected by masturbation (three to five days of ejaculatory abstinence) about one hour before insemination, to reduce the exposure time of spermatozoa to seminal plasma. After fluidification for 10-20 minutes at 37°C, semen samples were evaluated according to ^WHO criteria (2010)^. Semen with high viscosity were pretreated with the addiction of an equal volume of medium (G Gamete^TM^ Vitrolife).

### DG procedure

DG procedure was performed using three different gradient concentrations (95%, 70%, 50%) prepared with a density gradient solution (Spermient K-SISP COOK) and a buffer medium (G MOPS^TM^ Vitrolife). The 95% layer (0.5ml) was transferred into a 15-ml conical centrifuge tube (BD Falcon^TM^) and then the other layers (0.5ml) were gently released above the previous gradient. A volume of semen sample (0.5ml) was then placed on the top of the upper layer and the tube was centrifuged for 10 minutes at 1200rpm.

For each DG procedure, we used two 15ml conical tubes. After centrifugation, the lower layer was recovered in a sterile new tube and the pellet was resuspended with 5ml of G Gamete^TM^ Vitrolife for a subsequent centrifugation at 1200rpm for 5 minutes.

The supernatant was then removed and the pellet was submitted to swim-up by gently overlaying 1ml of G Gamete^TM^ Vitrolife. The tube was inclined at an angle of 45 degrees and left in the incubator for at least 40 minutes under controlled atmosphere conditions (6% CO_2_, 20% O_2_). After incubation, the supernatant was aspirated and transferred into a sterile new 15ml conical tube placed in the incubator until the time of injection. Sperm analysis was done using a sample fraction according to ^WHO criteria (2010)^.

### MSU procedure

After semen evaluation, about 1ml was recovered in a sterile 15ml conical tube and was submitted to a direct swim-up by gently overlying 1ml of G Gamete^TM^ Vitrolife. The tube was inclined at an angle of 45 degrees and left in the incubator until the time of injection under controlled atmospheric conditions (6% CO_2_, 20% O_2_). In this procedure, the use of the centrifuge was not expected. Sperm analysis was done using a fraction of the direct swim-up semen according to ^WHO criteria (2010)^. MSU procedure was performed at ICSI time according to the methods recently published (^Palini *et al.*, 2016^).

### Oocyte retrieval and embryo transfer

Ultrasound-guided oocyte retrieval was performed 36 hours after human chorionic gonadotropin (HCG) injection. Cumulus cells were removed 3 hours after pickup and the insemination procedure was performed 40 hours after HCG administration. Sibling oocytes were divided into two equal groups and inseminated by the same operator using spermatozoa obtained by DG and MSU. Approximately 1620 hours after insemination, fertilization was confirmed by the presence of two pronuclei and the extrusion of the second polar body (Istanbul Consensus 2011).

The embryos were cultured in a humidified atmosphere with 6% CO_2_, 5% O_2_, and remaining nitrogen; and on Day 3 (D3) they were classified according to the grading proposed by the ^ALPHA and ESHRE societies (Istanbul Consensus) (2011)^.

Elective fresh single embryo transfer (eSET) was performed on Day 5 (D5) after insemination; blastocyst cryopreservation was performed on Day 6 (D6) after insemination by Vitrification technique. D5 and D6 embryos were classified by all the Clinics according to a revisited Gardner grading (^Veeck & Zaninovic, 2003^).

Pregnancy was confirmed by serum beta HCG test 14 days after transfer.

### Statistical analysis

Differences between DG and MSU groups' numbers of mature and fertilized oocytes, numbers of D3 and D5/D6 embryos, and numbers of transferred and cryopreserved embryos were assessed by the t-test for paired data. Differences in fertilization and blastulation rates were assessed by the t-test for paired data after arcsine transformation of the data. Differences regarding pregnancy and abortion rates were evaluated by the Chi-square test. Probability values ≤0.05 were accepted.

## RESULTS

To test the effectiveness of MSU as compared to DG, the following indicators were considered: fertilization rate (FR), blastulation rate (BR), pregnancy rate (PR), and abortion rate (AR). Results are summarized in Table 1.

**Table 1:** Differences in fertilization rate (FR), blastulation rate (BR), pregnancy rate (PR), and abortion rate (AR) between DG and MSU groups.

	DG	MSU	P value
Mature oocytes	668	660	0.660
Fertilized oocytes	508	540	0.174
D3 embryos	460	496	0.132
D5/D6 embryos	212	316	<0.001
Transferred D5/D6 embryos	66	74	0.666
Cryopreserved D5/D6 embryos	82	168	<0.001
FR (%)	76.0 (508/668)	81.8 (540/660)	0.248
BR per fertilized oocyte (%)	41.7 (212/508)	58.5 (316/540)	0.009
BR per D3 embryo (%)	46.1 (212/460)	63.7 (316/496)	0.045
PR (%)	25.8 (17/66)	41.9 (31/74)	0.045
AR (%)	29.4 (5/17)	12.9 (4/31)	0.161

Differences in fertilization rates were not significant between the DG and MSU groups (76.0% vs. 81.8%, respectively, *p*=0.248); while significant differences were found for blastulation rates per fertilized oocyte (41.7% vs. 58.5%, *p*=0.009), blastulation rates per D3 embryos (46.1% vs. 63.7%, *p*=0.045), and pregnancy rates (25.8% vs. 41.9%, *p*=0.045). The abortion rate was reduced in the MSU group as compared to the DG, but not in a significant manner (12.9% vs. 29.4%, *p*=0.161), probably due to the small size of the population. Differences in the numbers of frozen D5/D6 embryos were significant between DG and MSU (82 vs. 168, *p*<0.001).

## DISCUSSION

A wide variety of sperm preparation protocols are currently available in IVF treatments. The most used techniques are density gradient (DG) centrifugation and swim-up (SU) in cases of normospermia or moderate oligoasthenoteratozoospermia. In patients with severe oligoasthenoteratozoospermia, strategies involving high speed centrifugation are used to recover spermatozoa from all semen samples.

The presence of a wide variability in the techniques used for semen treatments leads to a need to evaluate the potential benefits and risks of each procedure. The literature does not show a conclusion regarding fertilization and pregnancy rates in SU and DG procedures but it demonstrates benefits in favor of SU in terms of DNA damage and ROS production (^Zini *et al.*, 2000^).

The MSU technique used in the present study is similar to that of SU, but it offers the advantage of using no centrifugation, and still enabling an efficient selection of spermatozoa by both the SU into the conical tube and the MSU in polyvinylpyrrolidone (PVP), providing a gentler and safer treatment. The present study not only demonstrates the greater reproducibility of the results with MSU when compared with the DG, but what stands out is the possibility to use a non-invasive method on semen treatment with statistically better results for embryo development and pregnancy, that can potentially further increase, considering the cumulative pregnancy with subsequent embryo warming. Differences in the number of frozen D5/D6 embryos were significant between DG and MSU, probably due to the better quality of embryos obtained from the MSU group.

One explanation for this could be found in the better health status of the embryos obtained from spermatozoa that have suffered less stress during the treatment; in fact, paternal contribution to embryo genome could be evident, especially in blastocyst development (^Simon *et al.*, 2014^). Evidence of this is given by our preliminary results (data not shown) that demonstrate, through an array of Comparative Genetic Hybridization (aCGH) analysis, that blastocysts belonging to the MSU group have a better euploid status than those from the DG group. More data is needed to confirm this hypothesis.

Furthermore, we must take into consideration the potential epigenetic modifications that serial centrifugations and ROS production can have on spermatozoa, which are at potentially greater risk because of their extended processing specially during the DG technique. One study demonstrated a relationship between differential levels of sperm DNA methylation and pregnancy rate in IVF treatments (^Benchaib *et al.*, 2005^); a later study reported correlations between sperm DNA methylation and semen quality (^Houshdaran *et al.*, 2007^). Because of this, spermatozoa may be carriers of epigenetic modifications that may open another "epigenetic programming window" in the embryo, introducing changes in DNA imprint (^Miller *et al.*, 2014^).

It may be noted that the proposed MSU technique, while being safer, does not allow sperm capacitation (^Palini *et al.*, 2016^). However, our data shows that sperm activation through immobilization before ICSI can be enough to enable the fertilization process. Indeed, sperm plasma membrane damage has been described as a necessary process prior to ICSI, as it plays a key role in oocyte activation caused by spermatozoa (^Dozortsev *et al.*, 1995^; ^Palermo *et al.*, 1996^).

The SU procedure and the subsequent MSU also allowed to avoid the risk of mismatches, because they reduced the number of conical tubes used for the treatment of seminal samples. By comparing the two procedures used in the present study, a total of 3 tubes were used in DG, while only one after semen fluidification in MSU. Despite this, the presence of two operators was always required for a double check during the transition from the collecting container to the conical tube and from the conical tube to the ICSI dish (^Magli *et al.*, 2008^).

Another point in favor of SU and MSU treatments is the reduction of time and costs. In fact, sperm collection and SU can be performed in proximity of the ICSI insemination time, avoiding the risk of reduced DNA longevity. Unnecessary incubation of spermatozoa prior to artificial insemination or *in vitro* fertilization should be avoided, since sperm DNA longevity is significantly reduced after *ex vivo* sperm handling, and sperm DNA longevity after DG is lower when compared to sperm recovered from noncentrifuged semen (^Gosálvez *et al.*, 2014^). When using MSU, 30 minutes are sufficient for SU and 3 minutes for micro swim-up in PVP.

By using MSU, it is also possible to reduce costs since less consumables (such as pipettes and tubes), culture medium and gradient solution are required. Furthermore, although the insemination technique (ICSI) remains expensive for the need of a micromanipulator, it would be possible to insert the MSU procedure into an IVF low cost program, thus reducing the total cost of the treatment (^Van Blerkom *et al.*, 2014^; ^Teoh *et al.*, 2014^).

In conclusion, procedures that minimize excessive manipulation of sperm and centrifugation should be used in IVF treatment as a valid alternative to improve the outcome. This non-invasive technique enables to obtain embryos with a physiological and probably better euploid status, although further data is needed to prove it. By using the MSU procedure, it is also possible to reduce costs and mismatch errors because less steps are required for semen treatment, facilitating the workflow in an IVF laboratory.
